# Evolutionary Characterization of the Short Protein SPAAR

**DOI:** 10.3390/genes12121864

**Published:** 2021-11-24

**Authors:** Jiwon Lee, Aaron Wacholder, Anne-Ruxandra Carvunis

**Affiliations:** 1 Department of Computational and Systems Biology, School of Medicine, University of Pittsburgh, Pittsburgh, PA 15213, USA; jil303@pitt.edu (J.L.); acw87@pitt.edu (A.W.); 2 Pittsburgh Center for Evolutionary Biology and Medicine, School of Medicine, University of Pittsburgh, Pittsburgh, PA 15213, USA; 3 Joint CMU-Pitt Ph.D. Program in Computational Biology, University of Pittsburgh, Pittsburgh, PA 15213, USA

**Keywords:** microproteins, gene annotation, homology detection, *de novo* gene birth, protein evolution, adaptation, lncRNAs, noncanonical translation, comparative genomics, mTOR pathway

## Abstract

Microproteins (<100 amino acids) are receiving increasing recognition as important participants in numerous biological processes, but their evolutionary dynamics are poorly understood. SPAAR is a recently discovered microprotein that regulates muscle regeneration and angiogenesis through interactions with conserved signaling pathways. Interestingly, SPAAR does not belong to any known protein family and has known homologs exclusively among placental mammals. This lack of distant homology could be caused by challenges in homology detection of short sequences, or it could indicate a recent *de novo* emergence from a noncoding sequence. By integrating syntenic alignments and homology searches, we identify SPAAR orthologs in marsupials and monotremes, establishing that SPAAR has existed at least since the emergence of mammals. SPAAR shows substantial primary sequence divergence but retains a conserved protein structure. In primates, we infer two independent evolutionary events leading to the *de novo* origination of 5′ elongated isoforms of SPAAR from a noncoding sequence and find evidence of adaptive evolution in this extended region. Thus, SPAAR may be of ancient origin, but it appears to be experiencing continual evolutionary innovation in mammals.

## 1. Introduction

The human genome, as annotated in reference genome databases like RefSeq [1] and Ensembl [2], contains around 20,000 protein-coding genes. Developments over the last decade, however, suggest that eukaryotic genomes contain considerably more protein-coding sequences than annotated in genome databases [3]. Ribosome profiling and proteomic studies demonstrate widespread translation outside of annotated coding sequences, mostly predicted to generate short “microproteins” (<100 amino acids) [4,5,6,7,8,9,10]. Several microproteins identified by these techniques have been experimentally characterized and play key roles in biological pathways [4,11,12,13,14].

The evolutionary origins of microproteins are of considerable interest. Genome-wide studies find that the unannotated coding sequences identified by ribosome profiling tend to be much less conserved across species than annotated genes [7,15,16,17]. Among well-characterized microproteins, many lack homologs beyond a narrow taxonomic range. For example, the muscle performance regulator myoregulin [11], the muscle development microprotein Minion [18], and the mRNA decapping complex microprotein NoBody [19], each has predicted homologs in placental mammals but not in any other lineage. The lack of evident distant homology could indicate a recent *de novo* origin from a noncoding sequence [7,20,21,22], or may reflect homology detection failure due to the short length or rapid evolutionary divergence [23]. It is currently unclear whether recently discovered microproteins constitute a pool of previously unappreciated evolutionary molecular innovations or correspond to an ancient subset of the proteome that is only now coming to light. An in-depth characterization of the evolutionary history of microproteins is needed.

**Figure 1:**
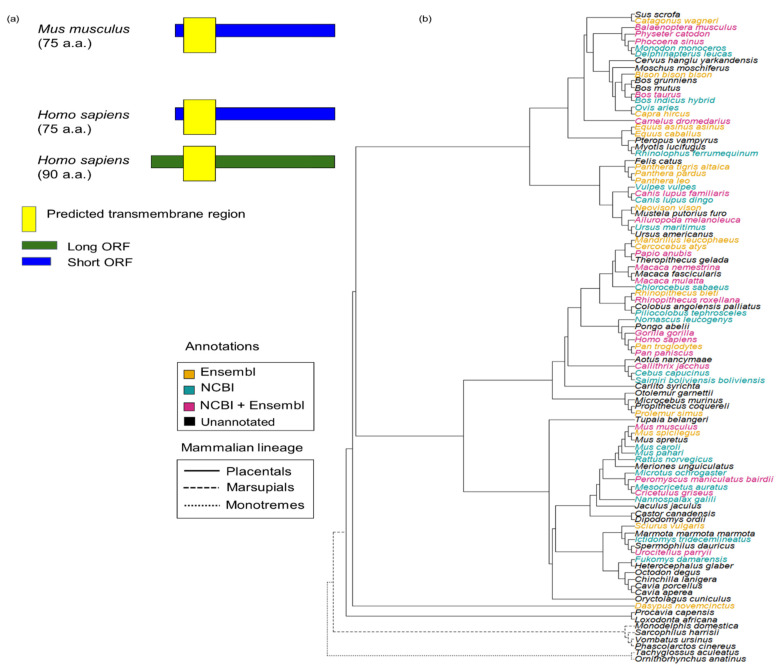
Current annotations of SPAAR are only present in placental mammals. (**a**) Only mouse and human SPAAR are experimentally validated [24]. Mouse has only a short isoform 75 a.a. long while humans have both this short isoform and a long isoform 90 a.a. long. A transmembrane region (yellow box) is predicted to be present near the N terminus of SPAAR [24]. (**b**) Species tree showing SPAAR annotations present in the Ensembl version 104 and NCBI Gene (August 2021 version) databases. SPAAR orthologs are predicted to exist only in placental mammals according to Ensembl and NCBI annotations. Tree structure was taken from Ensembl Compara [2]. Species were colored according to their annotation status of SPAAR in Ensembl version 104 and NCBI Gene (August 2021 version).

Here, we conducted an in-depth evolutionary analysis of the recently discovered mammalian microprotein SPAAR (initially reported as SPAR) [24]. SPAAR is translated from a transcript previously annotated as a lncRNA. SPAAR impedes mTORC1 [25] activation through interaction with v-ATPase [24]. It is downregulated upon acute injury, enhancing mTORC1 activity in muscle regeneration [24]. Recently, Spencer et al. reported a bifunctional role of the *SPAAR* locus, with the SPAAR microprotein and the previously annotated lncRNA that encodes it mediating opposing effects on angiogenesis through physical interactions with different proteins [26]. Two isoforms of the SPAAR protein have been experimentally characterized: a short form present in human and mouse, and a long form present in human but not in mouse (Figure 1a). SPAAR is currently only annotated in placental mammals. Our primary aim was to determine whether SPAAR is of recent *de novo* origin or is ancient and has homologs outside of placental mammals.

## 2. Materials and Methods

### 2.1. Initial Homology Search

The NCBI Gene [27] (version updated August 2021) and Ensembl release 104 [2] databases were queried to assess which species had annotations of SPAAR homologs. To identify additional homologs, the NCBI non-redundant protein sequences database (NR) was searched using BLASTP [28] and the non-redundant nucleotide collection (NR/NT) database was searched with TBLASTN [29] (database versions January 2021), using the 90 amino acid human protein sequence as query. An E-value of less than 10^−3^ was taken as the significance threshold. 

### 2.2. Syntenic Alignments

#### 2.2.1. Curation of Precomputed LastZ Alignments

A total of 310 pairwise LastZ [30] whole genome alignments with the homo sapiens GRCh38 genome assembly as reference were downloaded from the Ensembl Compara [2] FTP server on 26 July 2021. We curated these alignments and eliminated some from further analyses according to the following criteria. First, genome assemblies that were listed as projection builds were removed. Second, the genomes of species that were included in neither one of the two species trees precomputed in Ensembl version 104 [2] were also removed. Third, the most recent genome assembly was selected for species that had multiple pairwise alignments available. Finally, four additional genomes were removed from consideration: the gibbon, Bolivian squirrel monkey, and panda genomes were removed because the genome assemblies used for LastZ alignments exhibited numerous mismatches at the SPAAR locus with the current genome assemblies used for annotations in Ensembl and NCBI; the Argentine black and white tegu reptile was removed because we detected transposable elements specific to the mammalian lineage in the genome region aligned to the *SPAAR* locus, indicating a possible contamination. This curation resulted in limiting the set of LastZ whole genome alignments assessed in further analyses to 248 assemblies (Table S1).

#### 2.2.2. Identification of SPAAR ORFs from Syntenic Alignments

Pairwise blocks corresponding to coordinates of the SPAAR exon ENSE00001789136 were extracted from the curated pairwise LastZ alignments. Coordinates for ENSE00001789136 were retrieved from the Ensembl database via the R/Bioconductor package BioMart version 2.48.3 [31]. For all assembled genomes, the region of the pairwise alignment aligned to the human 75 a.a. *SPAAR* open reading frame (ORF) was extracted from the Ensembl LastZ alignments. Presence of the ORF in the comparison species was confirmed if it had a start codon aligned to the human start, an in-frame stop codon aligned to the human stop, and no intermediate stop codons. We also considered the ORF presence confirmed if the first in-frame stop was identified after the region aligned to the human stop, or if the first detected start codon in the alignment was in frame with a stop codon aligned to the human stop.

#### 2.2.3. Identification of Unannotated HRCT1 Orthologs

HRCT1 orthologs were first searched for in LastZ pairwise alignments, following the same procedure outlined in 2.2.1 for SPAAR. To find a monotreme ortholog, we queried a profile hidden Markov model of a mouse, human, wombat, and Tasmanian devil HRCT1 nucleotide multiple sequence alignment (MSA) against the platypus whole genome assembly with HMMER version 3.3.2 [32], using the command nhmmer with default options. Additional marsupial and monotreme orthologs were searched for using the orthologs identified by these procedures as queries in TBLASTN searches of genome assemblies.

### 2.3. Gene Expression Analysis

The NCBI Sequence Read Archive [33] was searched for RNAseq and Ribo-seq data in marsupials and monotremes. RNAseq reads from two studies with transcriptomics data from human, mouse platypus, and opossum [34,35] (accession: SRP102989, ERP111066) were mapped to each species’ genome (Table S2) with HISAT2 version 2.2.2 [36], restricting the mapping to strand-specific samples, after trimming adapter sequences and removing low quality reads with Trim Galore version 0.6.5 [37]. The density of reads spanning genomic coordinates of the SPAAR ORF was visually assessed in IGV [38] for evidence of transcription in each species. StringTie version 2.1.6 [39] was used to assemble transcripts and predict transcript architecture from mapped HISAT2 reads of heart tissue in each species. Ribo-seq reads from Wang et al. [35] (ERP111066) were mapped to the platypus and opossum genome using the STAR read aligner [40]. Reads were then remapped to the ribosome *p*-site by examining Ribo-seq read patterns of annotated genes. To accomplish this, all reads were shifted such that a read pattern of triplet periodicity corresponded to the coding sequence of annotated genes, as described in Malone et al. 2017 [41]. The significance of triplet periodicity of Ribo-seq reads was then assessed for the SPAAR ORF in each tissue using the method described in Wacholder et al. [16]. For each codon, the position within the codon that had the most reads was determined. A binomial test that the number of codons in which the first position had the most reads was greater than 1/3, among all codons where a single position had the most reads, was run to obtain a *p*-value.

### 2.4. Remote Homology Detection

SPAAR sequences of human, mouse and the marsupials and monotremes found through LastZ alignments or TBLASTN were queried against non-mammalian genome assemblies (listed in Supplementary Table S3) with TBLASTN. Annotated human and mouse SPAAR exons, and predicted exons in platypus and opossum were also queried against these genomes with discontiguous MegaBLAST [42]. PSI-BLAST [29] was run on the 90 amino acid human protein sequence. A selection of SPAAR protein sequences were queried against the Pfam database [43] to assess possible emergence from distantly related protein families, and HMMER [44] was used to query MSA profiles (hmmsearch) and single protein sequences (phmmer) on the HMMER webserver version 2.40 [44]. The 605-way combined mammalian and avian Cactus alignment [45] was also checked for avian species aligned to the SPAAR region.

### 2.5. Conservation Analyses in Mammalian Lineages

#### 2.5.1. Multiple Sequence Alignments and Guide Trees

Multiple sequence alignments were performed with MAFFT L-INS-i through the MAFFT online service [46], using default parameters. Guide trees were generated by first pruning a precomputed species tree from Ensembl Compara [2] to relevant species branches using the ape R package version 5.5 [47]. The pruned species tree and the MAFFT alignment were then used to generate the guide tree with PhyML version 3.1 [48] using the command “phyml -d nt -m HKY85 -v e -o lr -c 4 -a e -b 0 -f e -u (species tree)”.

#### 2.5.2. Conservation Analyses in Mammalian Lineages

Pairwise dN/dS and selection tests were performed using codeml in the PAML software [49]. Pairwise alignments were used to estimate dN/dS in placentals (mouse, human) and monotremes (platypus, echidna), and an MSA generated following 2.5.1 was used to assess marsupial sequences (wombat, Tasmanian devil, koala, opossum). The “F1 × 4” codon frequency option with runmode “pairwise” was used, and for marsupials, a guide tree generated following 2.5.1 was also input in the dN/dS analyses.

### 2.6. Structural Predictions

TMHMM version 2 [50] was used to predict the positions of transmembrane domains, and disordered residues were predicted with Disopred version 2.43 [51] through the Robetta server [52]. Protein structural predictions based on amino acid sequences were performed with the AlphaFold2 [53] Colab notebook. Default settings were used for all software. PyMOL version 2.5.2 [54] was used for the visualization of structures.

### 2.7. Analyses of Long SPAAR ORF

#### 2.7.1. Addition of Primate Sequences

Sequences from three additional species that were omitted from the LastZ analysis (*Nomascus leucogenys*, *Pongo abelii*, *Saimiri boliviens boliviens*), were added to our analysis after finding updated genome assemblies to increase power (Table S4). BLASTN of the sequence spanning the start of the human SPAAR exon ENSE00001789136 to the end of the SPAAR ORF was used as query to find homologous sequences in the three species.

#### 2.7.2. Ancestral Sequence Reconstruction

Ancestral sequences for the primate lineage were constructed with PRANK version 0.170427 [55] using the parameters “-showanc -showevents –F” using a guide tree generated with PhyML as described in Section 2.5.1.

#### 2.7.3. Site-Specific Positive Selection Test

The codeml command in the PAML software [49] was used to assess sites under positive selection, with the “F1X4” codon frequency option. An MSA of 20 primates with the 90 a.a. SPAAR, and a guide tree generated following Section 2.5.1 were provided as input.

## 3. Results

### 3.1. Identification of SPAAR Orthologs Outside of Placental Mammals

To investigate the evolutionary history of SPAAR, we first assessed what was already known about SPAAR within genome databases. SPAAR is currently only experimentally validated in mouse and humans (Figure 1a). We searched for additional records of SPAAR orthologs in NCBI Gene [27] (version updated August 2021) and Ensembl release 104 [2]. Ensembl and NCBI Gene had 36 and 99 annotations of SPAAR orthologs, respectively (Figure S1). These annotations were restricted to the placental mammal lineage, with no annotations in marsupials, monotremes, or any other vertebrate (Figure 1b and Figure S1, Tables S5 and S6).

To search for additional SPAAR homologs, we first performed BLASTP and TBLASTN queries of the human SPAAR sequence (Materials and Methods—Section 2.1). Initial homology searches with BLASTP and TBLASTN returned only results in placental mammals. 

**Figure 2:**
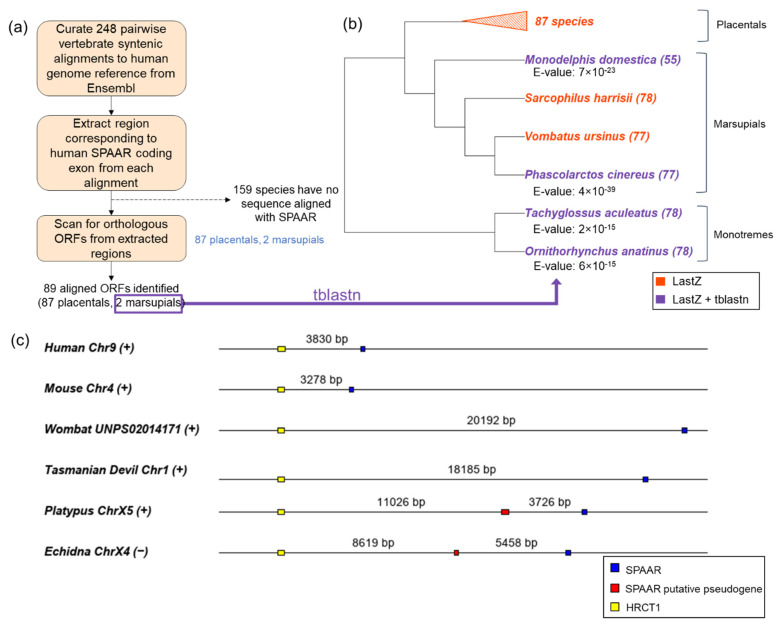
SPAAR orthologs are detected across all three mammalian lineages. (**a**) Schematic of ORF detection through LastZ alignments. LastZ alignments allowed us to detect SPAAR orthologs in 87/87 placentals and in 2/4 marsupials in our curated set of vertebrate syntenic alignments. (**b**) SPAAR orthologs were further detected throughout marsupials and monotremes by TBLASTN searches using the marsupial sequences detected through LastZ alignments as query sequences. Lowest TBLASTN E-values for each ortholog are listed below the species names. The length of detected ORFs (amino acids) are indicated in parentheses after the species names. (**c**) Positions of SPAAR mapped to genomic coordinates show preservation of microsynteny across mammalian lineages. Distances between each open reading frame are to scale. SPAAR homologous regions are shown in blue, a putative pseudogenic region to SPAAR in red, and HRCT1 in yellow. (+) and (−) beside species names indicate sense and anti-sense orientation on the chromosome.

For more sensitive detection of homologous sequences, we curated and analyzed 248 vertebrate pairwise Ensembl LastZ whole genome alignments to the human GRCh38 genome assembly (Figure 2a, Materials and Methods—Section 2.2). Of the 248 alignments assessed, 159 species did not have any sequence that was aligned to the human *SPAAR* coding exon (Table S1). All species that had sequences aligned to *SPAAR* in the LastZ alignments had an intact homologous open reading frame (ORF) at the locus (Figure 2a): homologous ORF sequences to SPAAR were detected in two marsupial species, the common wombat and Tasmanian devil, in addition to all 87 placental mammals assessed (Table S7). These ORFs corresponded to the SPAAR homologs predicted by public databases for 53 of the 87 placental species, and to unannotated ORFs for the two marsupial species and 34 placental species.

To search for more SPAAR orthologs outside of placental mammals, we used the two marsupial ORF sequences found through the pairwise LastZ alignments as TBLASTN queries against whole genome assemblies of two other marsupials (koala and opossum) and two monotremes (platypus and echidna). Sequences homologous to SPAAR were found in all four marsupial and monotreme species assessed (TBLASTN E-values < 6 × 10^−15^, Figure 2b, Table S2), and further analyses confirmed the presence of intact homologous ORFs (Materials and Methods—Section 2.2.2). Thus, our findings expand the phylogenetic range of SPAAR relative to current annotations and reveal that homologs of SPAAR exist in all three major extant mammalian groups.

To determine whether the homologous ORFs identified by TBLASTN are one-to-one orthologs of human *SPAAR*, we sought to assess whether syntenic relationships were maintained between *SPAAR* and its nearby gene, *HRCT1*, across placentals, marsupials and monotremes. However, *HRCT1*, only has placental mammal orthologs predicted in Ensembl [2]. We thus first searched for unannotated homologs of *HRCT1* in marsupials and monotremes (Materials and Methods—Section 2.2.3). Having identified homologs of both *SPAAR* and *HRCT1*, we examined their genomic coordinates and confirmed that *HRCT1* and *SPAAR* had maintained gene order across all mammalian lineages (Figure 2c) despite substantial rearrangements undergone by the human chromosome 9 throughout its evolutionary history (Figure S2). An additional sequence with a significant TBLASTN match to the Tasmanian devil *SPAAR* was found to be located in between *HRCT1* and the strongest *SPAAR* match in both monotremes, with a truncated length relative to *SPAAR* in the echidna and elongated length relative to *SPAAR* in the platypus. These sequences were substantially weaker TBLASTN matches to the Tasmanian devil *SPAAR* (E-values: 2 × 10^−9^ vs. 6 × 10^−15^ in platypus; 5 × 10^−4^ vs. 2 × 10^−15^ in echidna), suggesting they may be pseudogenes. The persistence of synteny between *SPAAR* and *HRCT1* is strong evidence of vertical descent. Thus, the unannotated ORFs we identified in marsupials and monotremes (Figure 2b) are orthologous to *SPAAR.*

**Figure 3:**
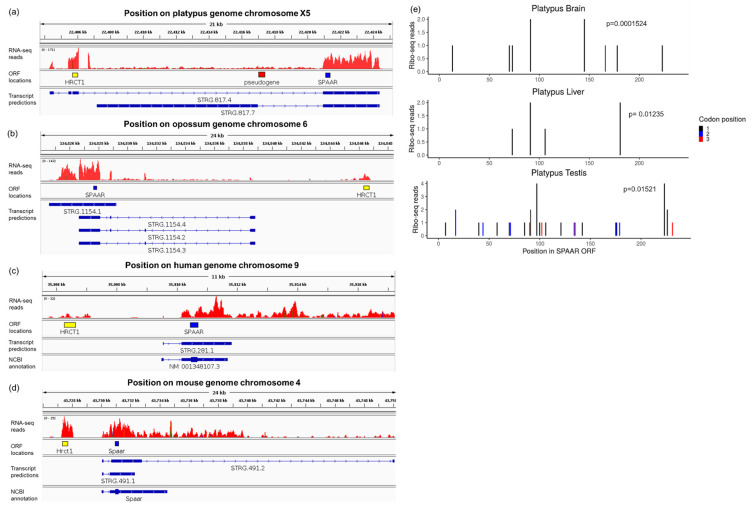
SPAAR is expressed in marsupials and monotremes. (**a**–**d**) RNA-seq reads of platypus, opossum, human, and mouse heart tissue as displayed in IGV [38]. Data were from Marin et al. [34]. The locations of ORFs encoding HRCT1, a putative SPAAR pseudogene, and SPAAR have been highlighted in yellow, red, and blue, respectively, for ease of comparison across the locus. The transcript predictions derived from the RNA-seq reads displayed, as well as the NCBI annotations for human and mouse, are represented in blue. Only transcripts models overlapping the SPAAR coding sequence are shown. The arrows within the transcript models represent the direction of transcription, on the positive strand for platypus human and mouse, and on the negative strand for opossum. (**e**) Ribo-seq reads of platypus in brain, liver, and testis. Data from Wang et al. [35]. The *x*-axis corresponds to each position in the 237 bp long SPAAR ORF in the platypus, with 1 as the first position. Reads in each platypus tissue show significant triplet periodicity by a binomial test [16], with a preference for aligning to the first codon position shown in black lines.

Nongenic sequences contain numerous nongenic ORFs that are not expressed into proteins [4]. To determine if the marsupial and monotreme *SPAAR* orthologous ORFs are transcribed, we mapped RNA-seq data generated from multiple tissues of a marsupial species (opossum) and a monotreme species (platypus) by two studies [34,35] to genomic positions (Materials and Methods—Section 2.3). We observed a dense region of reads spanning the *SPAAR* orthologous ORF in both species (Figure 3a,b and Figure S3). Transcript assemblies further supported the presence of long multi-exonic transcripts containing the SPAAR ORF in the opossum and the platypus (Figure 3a,b), similar to human and mouse (Figure 3c,d) (Table S8, Materials and Methods—Section 2.3). Interestingly, transcript models for platypus indicate that the SPAAR and HRCT1 coding sequences sometimes are on the same transcript, which could in part explain their conserved syntenic linkage. To search for evidence of translation, we mapped Ribo-seq data generated for platypus and opossum in three tissues (testis, brain, and liver) [35] to genomic positions (Materials and Methods—Section 2.3). No read mapped to the *SPAAR* locus in the opossum data set, but the platypus data set contained a small number of reads spanning the *SPAAR* locus. These reads nevertheless indicated evidence of translation in the platypus as they showed the significant triplet periodicity characteristic of codon-by codon progression of ribosomes (*p* < 0.05; Figure 3e, Materials and Methods—Section 2.3). Altogether, these results demonstrate that *SPAAR* orthologs are expressed in marsupials and monotremes. The identification of long SPAAR-encoding transcripts in monotremes and marsupials suggests that the bifunctional lncRNA-like and mRNA role of the SPAAR transcript that has been demonstrated in human and mouse [26] may also exist throughout mammals. Altogether, homology data and gene expression data support the proposition that the *SPAAR* gene is at least as ancient as the mammalian lineage.

To determine if we could further trace the ancestry of *SPAAR*, we next conducted an extensive search for *SPAAR* homologs outside of mammals. We first extended our TBLASTN analysis, searching for homologs using the identified marsupial and monotreme SPAAR protein sequences as queries in addition to human and mouse SPAAR. However, no significant TBLASTN matches were observed in a collection of non-mammalian animal genomes (Table S3). We next attempted to use the conserved synteny between SPAAR and HRCT1 to identify the corresponding genomic region in non-mammalian species, but we were also unable to find matches to HRCT1 outside mammals by TBLASTN. To determine if we could identify homologs to the *SPAAR* transcript, we then used discontiguous MegaBLAST [42], using annotated human and mouse exons, and all predicted exons in opossum and platypus (Table S8) as queries against non-mammalian genomes, but again identified no matches after excluding hits to transposable element sequences [56]. Finally, we tried several additional algorithms designed for sensitive remote homology detection, including PSI-BLAST [29] and HMMER [44], but found no conclusive results (Figure S4, Materials and Methods—Section 2.4). Altogether, these analyses did not allow us to determine whether SPAAR has evolved *de novo* in mammals or derives from an older gene that underwent extreme sequence divergence in mammals preventing identification of distant homologs. 

### 3.2. Sequence Divergence and Structural Conservation of SPAAR Orthologs

The SPAAR orthologs we identified throughout all three mammalian lineages (Figure 1, Figure 2 and Figure 3) had been missed by automatic genome annotation pipelines because of extensive primary sequence divergence. To investigate the consequences of sequence divergence on the SPAAR protein, we examined SPAAR conservation at the amino acid and structural level. Pairwise dN/dS analyses within each of the three mammalian lineages are shown in Table 1 (Section 2.5). All pairwise dN/dS calculations were <0.5, suggesting that the SPAAR protein sequence is evolving under purifying selection within each lineage.

**Figure 4:**
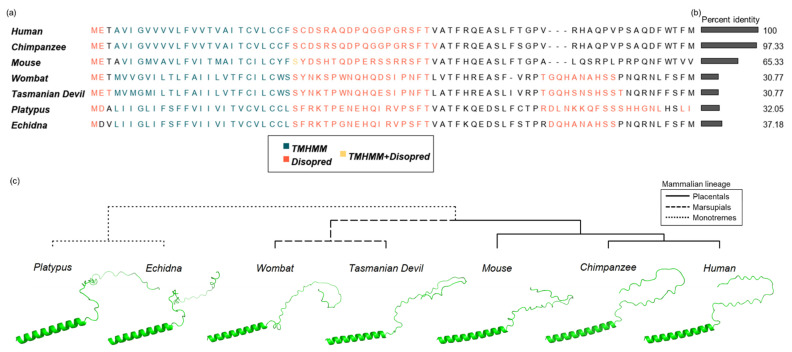
Structural predictions across SPAAR orthologs predict consistent transmembrane and disordered regions. (**a**) Multiple sequence alignment of 7 SPAAR orthologs, with predictions of disordered residues and transmembrane domains. Predicted transmembrane domains as predicted with TMHMM [50] are shown in teal, disordered residues as predicted by Disopred [51] in orange, and positions with both transmembrane domain and disorder predictions are shown in yellow. (**b**) Amino acid identity of each ortholog to the human SPAAR sequence is shown. (**c**) Structural predictions of SPAAR orthologs, as generated by AlphaFold2 [53] arranged in a cladogram.

*De novo* structural predictions of SPAAR orthologs also showed preservation of structure across mammals, with a consistent prediction of a 23-amino-acid transmembrane domain near the N-terminus, and an 18–19-amino-acid disordered region (protein regions that do not converge onto one structural conformation) immediately following the transmembrane domain (Figure 4a, Section 2.6). This level of structural conservation contrasted with low amino acid identity when comparing monotreme or marsupial orthologs to human SPAAR (Figure 4b). An additional region of disorder was detected in marsupial and monotreme sequences closer to the C-terminus (Figure 4a). No additional secondary structures were predicted with AlphaFold2 (Figure 4c). Thus, our analyses suggest that SPAAR has maintained the basic structure of a transmembrane domain followed by a disordered region throughout the mammalian lineage despite substantive sequence divergence.

### 3.3. Emergence of Long SPAAR Isoform from Noncoding Sequences in Primates

**Figure 5:**
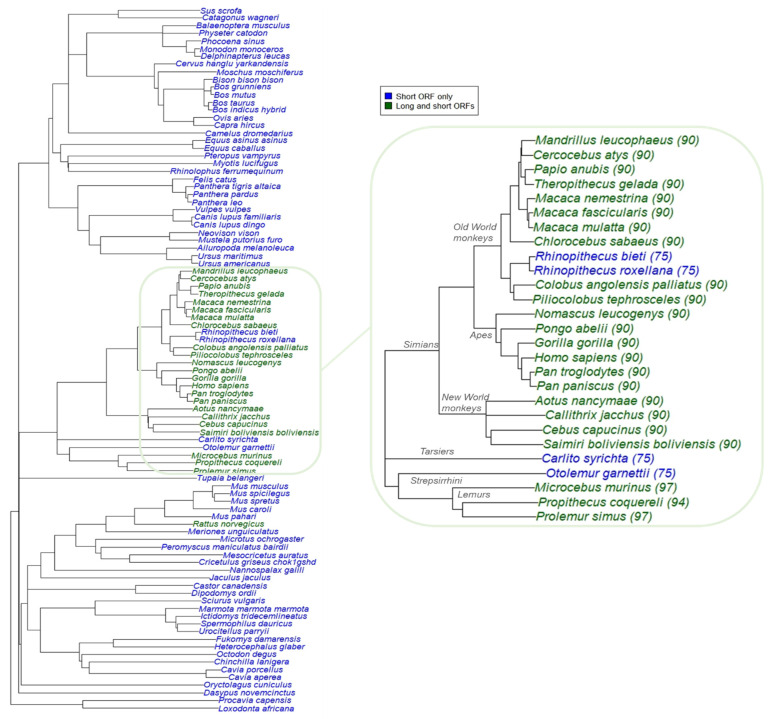
Detection of long SPAAR in placental mammals suggest occurrence of elongation event in primate lineage. Left: species tree of 91 placental mammals with SPAAR ORFs. Species include 87 placentals assessed in LastZ analysis, *Homo sapiens*, and three additional primate species (*Nomascus leucogenys*, *Saimiri boliviensis boliviensis*, and *Pongo abelii*) with updated genome assemblies (Table S4). Species in blue were found to have an intact short SPAAR ORF only, and species in green were found to have both long and short ORFs. Right: same data, zooming onto the primate phylogeny with long ORF amino acid lengths in parentheses.

Previous experimental research showed that humans have both a long and a short isoform of SPAAR while mice only have a short isoform (Figure 1a). The long human isoform is less expressed, and has a weaker impact on mTORC1 inhibition, than the short isoform [24]. We investigated the evolutionary history of this long isoform and its possible biological significance. To identify which species other than humans may have a longer isoform, we searched upstream of the region aligned to the human-annotated *SPAAR* short isoform in all 87 placental mammal alignments from our LastZ analysis for possible additional in-frame start codons (Materials and Methods—Section 2.2.2). Besides *Rattus norvegicus*, which had an intact ORF that was 169 amino acids long, the only species with an intact long ORF (predicted protein length greater >80 a.a.) were within the primate lineage (Figure 5). All New World monkeys, Old World monkeys, and apes had a 90 amino acid sequence orthologous to the experimentally validated human long SPAAR, apart from the *Rhinopithecus* species. Three of four lemurs assessed also appeared to have a long ORF 94 or 97 amino acids in length. TMHMM and Disopred predictions for the 90 a.a. long ORF sequences also predicted consistent transmembrane and disordered region predictions that were almost identical to those of the short placental mammals in Figure 4a, but the primates had an additional N-terminal disordered region absent from other mammals (Figure S5).

**Figure 6:**
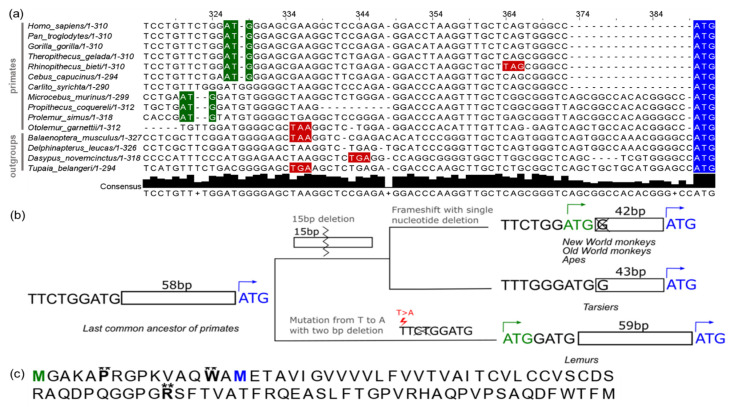
Long SPAAR ORF emerged twice independently from nongenic sequences in the primate lineage. (**a**) Multiple sequence alignment of a selection of primate species and four outgroup species. Start codons of the short SPAAR are highlighted in blue; start codons that are in frame with the short SPAAR start codon are highlighted in green; in-frame stop codons upstream of the short SPAAR are highlighted in red. Alignment was performed using MAFFT. (**b**) Schematic showing critical mutations that led to the emergence of the long SPAAR ORF in primates. A mutation from T to A, with a two nucleotide deletion and one nucleotide insertion led to a start codon being in frame with the short SPAAR ORF in the lemur lineage, and a 15 bp deletion followed by a frameshift mutation resulting from a single nucleotide deletion resulted in an upstream start codon emerging in New World monkeys, Old World monkeys, and apes. Ancestral reconstruction was performed with PRANK; an MSA of all ancestral sequences is shown in Figure S6. (**c**) Human long SPAAR protein sequence with sites significant for Naïve Empirical Bayes positive site specific selection (** probability > 0.99). Two sites between the long and short SPAAR start codons were significant, with calculated mean ω of 1.798 for the proline at the sixth position, and mean ω of 1.799 for the tryptophan at the fourteenth position. The methionines corresponding with the long and short SPAAR start codons are shown in green and blue, respectively. A MAFFT MSA of 20 primate sequences with a 90 a.a. ORF was used with PAML for this analysis.

To assess the evolutionary origins of the long SPAAR isoform, we generated an MSA covering the region of the protein present only in the long ORF (Figure 6a, Materials and Methods—Section 2.5.1). We then performed ancestral sequence reconstruction using PRANK [55], with sequences from 27 primate species (Materials and Methods—Section 2.7.2). Two deletions in the simian lineage brought an upstream ATG in-frame with the *SPAAR* coding sequence, generating an isoform containing a 15 amino acid extension. Separately, in the lemur lineage, a T to A mutation generated a new ATG in frame with the *SPAAR* coding sequence, creating a potential isoform with a 22 amino acid extension. Thus, ancestral sequence reconstruction shows that the long *SPAAR* ORF isoform emerged independently *de novo* from nongenic sequences in two different primate lineages (Figure 6b and Figure S6).

To investigate whether the evolutionarily novel elongation of SPAAR in primates may bear adaptive significance, we tested whether the 90 a.a. SPAAR protein contains sites undergoing positive selection. An MSA was generated from the protein sequences of 20 primate species with an intact 90 a.a. SPAAR ORF and tested for site specific positive selection using PAML (Materials and Methods—Section 2.7.3). These analyses revealed three sites with a high likelihood of site-specific positive selection when tested with the Naïve Bayes estimator (probability >0.99; ω = 1.798 and 1.799, respectively; Figure 6c). Two of these three sites were located in between the long and short SPAAR start codons (Figure 6c), suggesting that the *de novo* emerged N-terminal tail of primate SPAAR mediates molecular mechanisms with adaptive, primate-specific consequences.

## 4. Conclusions

Here, we investigated the evolutionary history of the recently discovered microprotein SPAAR [24]. Though SPAAR is currently annotated only in placental mammals, we demonstrate that SPAAR has orthologs in marsupials and monotremes as well. These orthologs are transcribed in long multi-exonic transcripts, translated, and evolving under purifying selection at the amino acid sequence and protein structure levels, confirming that they are conserved genes. While attempts at more distant homology detection did not yield clear matches, the short size of SPAAR and its high sequence divergence within mammals indicate that homology would be difficult to detect even if SPAAR emerged far earlier [23,57]. Thus, we can infer with high confidence that SPAAR and the long transcripts encoding it have existed at least since the emergence of mammals, and may be much older. 

Intriguingly, we identified two independent evolutionary events leading to a potential elongated SPAAR isoform in primates (of which the human isoform is confirmed to exist [24]). Our ancestral reconstruction analyses showed that this N terminal tail emerged *de novo* from previously nongenic sequences. Furthermore, we detected positive selection on sites within this N-terminal extension. These observations provide evidence of recent functional innovation in primates, though the specific role of the long SPAAR isoform remains to be discovered.

Analyses of taxonomically-restricted annotated genes suggest that a lack of detected distant homology is more often the result of *de novo* origin than homology detection failure [58]. Nevertheless, it is also clear that homology detection failure is common and expected for short coding sequences [23]. Despite these challenges, and the poor annotation of microproteins in existing genome databases, the SPAAR example demonstrates that thorough evolutionary analysis can readily expand the identified phylogenetic range even of a gene that is both short and rapidly evolving. New computational methods are needed to systematically identify and annotate microprotein orthologs. This will reveal whether microproteins follow the same evolutionary dynamics as the rest of the proteome or exhibit distinct patterns of protein evolution.

## Figures and Tables

**Table 1:** dN/dS across mammalian lineages.

Species 1	Species 2	N	S	dN	dS	dN/dS	*p*-Value
Human	Mouse	170.1	54.9	0.1795	1.2873	0.1395 (±0.0545)	5.4222 × 10^−7^ *
Koala	Wombat	167.4	57.6	0.0426	0.0997	0.4274 (±0.267)	4.177 × 10^−1^
Tasmanian Devil	Wombat	168	57	0.0545	0.3203	0.1701 (±0.0816)	1.004 × 10^−3^ *
Tasmanian Devil	Koala	166.9	58.1	0.061	0.4442	0.1373 (±0.0618)	3.410 × 10^−5^ *
Opossum	Wombat	168.3	56.7	0.0826	0.296	0.2789 (±0.1211)	1.707 × 10^−2^ *
Opossum	Koala	168.8	56.2	0.0881	0.3333	0.2642 (±0.1117)	8.962 × 10^−3^ *
Opossum	Tasmanian Devil	162.2	62.8	0.071	0.4726	0.1504 (±0.0639)	3.429 × 10^−5^ *
Platypus	Echidna	162.8	71.2	0.0523	0.1289	0.4061 (±0.2110)	2.481 × 10^−1^

* *p* < 0.05.

## Data Availability

All data used in this study are publicly available, and additional data generated through analyses of these datasets are available in Tables S7–S8. All pairwise LastZ alignments to the human genome version GRCh38 are available on the Ensembl Compara FTP server: http://ftp.ensembl.org/pub/current_maf/ensembl-compara/pairwise_alignments. RNAseq and Ribo-seq data used for analyses can be accessed at the NCBI Sequence Read Archive (https://www.ncbi.nlm.nih.gov/sra) with accession numbers SRP102989 and ERP111066. All genome assemblies used for analyses are listed with their GenBank genome accession numbers in Tables S1–S4 and can be accessed through NCBI GenBank (https://www.ncbi.nlm.nih.gov/genbank/). Species trees used to generate figures were downloaded from the Ensembl Compara Github page (https://github.com/Ensembl/ensembl-compara/blob/release/104/conf/benchmark/species_tree.branch_len.nw; https://github.com/Ensembl/ensembl-compara/blob/release/104/conf/vertebrates/species_tree.branch_len.nw).

## Supplementary Materials

The following are available online at https://www.mdpi.com/article/10.3390/genes12121864/s1, Figure S1: SPAAR is only annotated in placental mammals in Ensembl and NCBI Gene, Figure S2: Genomic region of SPAAR has undergone several chromosomal rearrangements, Figure S3: RNA-seq reads of platypus and opossum in SPAAR locus, Figure S4: Inconclusive remote homology detection results, Figure S5: Prediction of transmembrane and disordered regions across primate species with long SPAAR ORF, Figure S6: Ancestral reconstruction of SPAAR in primates with critical mutations leading to two elongations, Table S1: Genome assemblies assessed for presence of syntenic block to SPAAR, Table S2: SPAAR in marsupial and monotreme genome assemblies, Table S3: Non-mammalian genomes used to search for SPAAR, Table S4: Additional primate genomes used for assessment of long SPAAR ORF, Table S5: Annotations of SPAAR in NCBI Gene (28-August-2021), Table S6: Annotations of SPAAR in Ensembl release 104, Table S7: ORF sequences found through LastZ syntenic alignments, Table S8: StringTie SPAAR transcript predictions in opossum and platypus heart tissue.
