# Supplementary material for: Evolutionary Characterization of the Short Protein SPAAR
Source: Genes (Basel). 2021 Nov 24;12(12):1864. doi: 10.3390/genes12121864 (PMC8702040; doi:10.3390/genes12121864)
Supplement: Supplementary file 1 [file genes-12-01864-s001.zip › Supplementary Figures - revised.pdf]

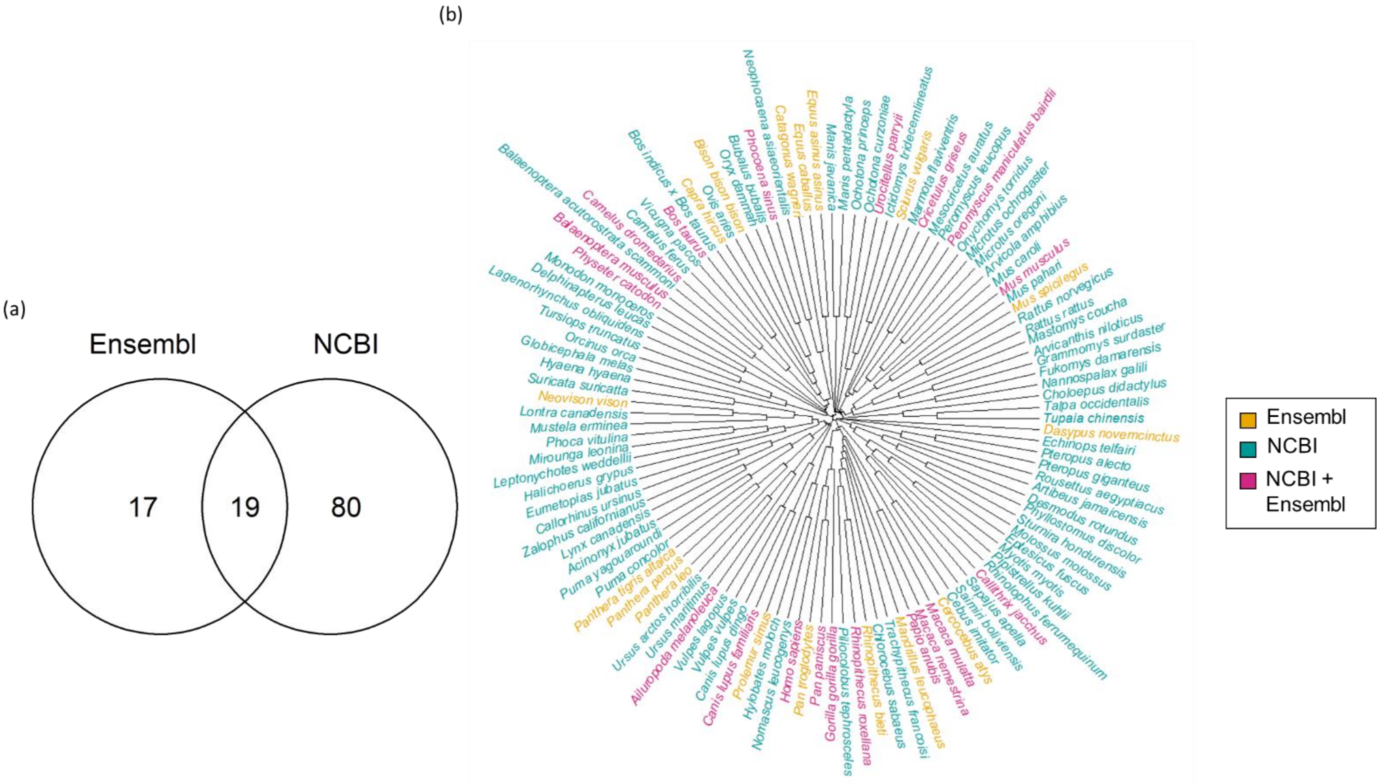

Figure S1. SPAAR is only annotated in placental mammals in Ensembl and NCBI Gene. (a) Venn diagram of placental mammal species annotated with SPAAR in Ensembl version 104 *[1]* and NCBI Gene (August 2021 version) *[2]*. (b) Cladogram of all placental mammal species found across Ensembl and NCBI Gene databases. Species were colored according to their annotation status of SPAAR in Ensembl version 104 and NCBI Gene (Aug 2021 version). Taxonomic structure was retrieved from NCBI Taxonomy *[3]*.

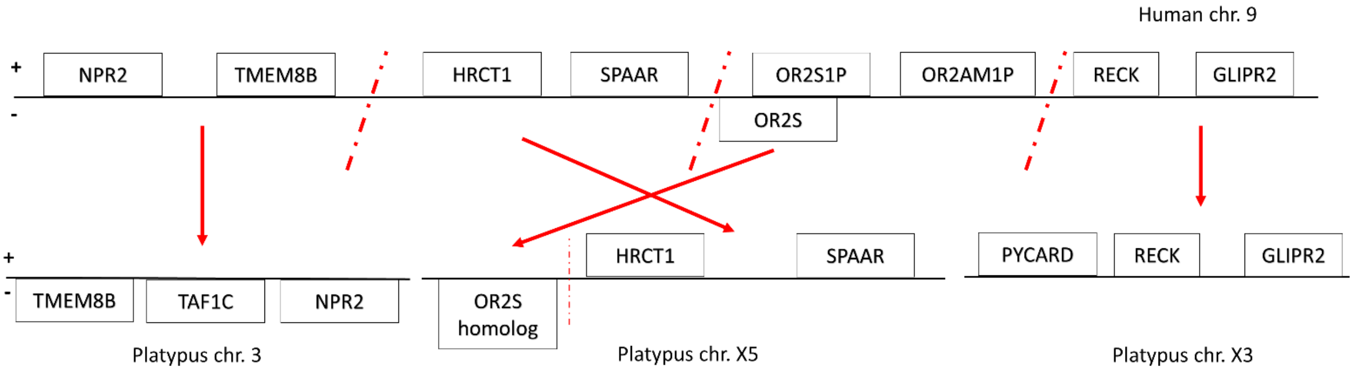

Figure S2. Genomic region of SPAAR has undergone several chromosomal rearrangements. Locations of genes in close proximity to SPAAR in human chromosome 9, and their corresponding locations in platypus are shown. Genes above each line reflect their position on the (+) strand of each chromosome assembly, and genes below each line reflect their position on the (-) strand of each assembly. Genomic coordinates for NPR2, TMEM8B, OR2S, OR2S1P, OR2AM1P, RECK, GLIPR2, PYCARD, and TAF1C, and for the human SPAAR and HRCT1 were retrieved from Ensembl version 104 [1]. Coordinates for HRCT1 and SPAAR in the platypus were identified in this study. The platypus OR2S homolog coordinates were identified through a tblastn [4] query of the human OR2S against the platypus genome assembly.

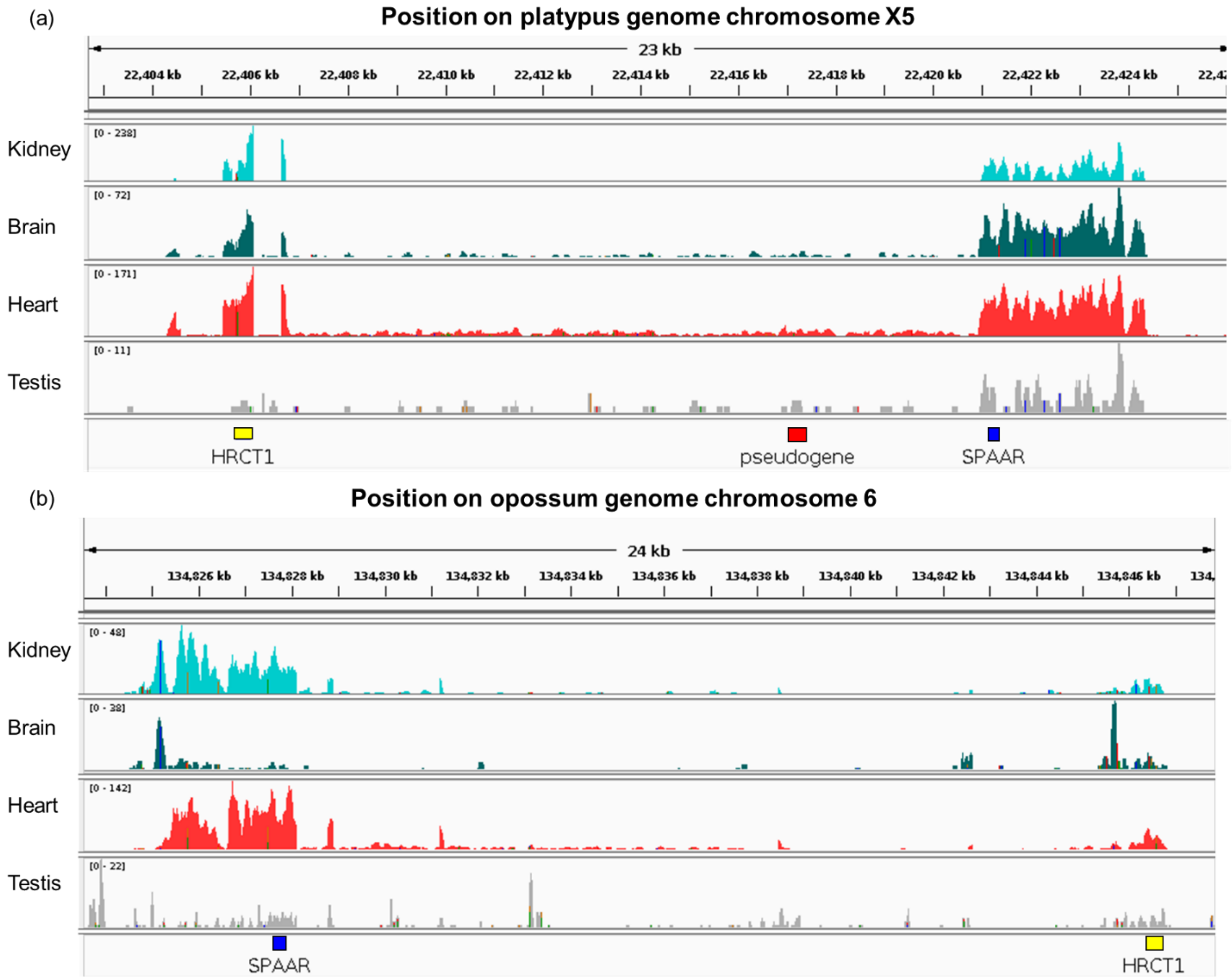

Figure S3. RNA-seq reads of platypus and opossum in SPAAR locus. (a-b) RNA-seq reads from platypus and opossum tissues as displayed in IGV [5]. Data displayed for platypus was from one study[6], and for opossum from two studies [6,7]. The locations of the HRCT1 ORF, putative SPAAR pseudogene, and SPAAR have been highlighted in yellow, red, and blue, respectively, to easily compare density of reads across the region.

(a)

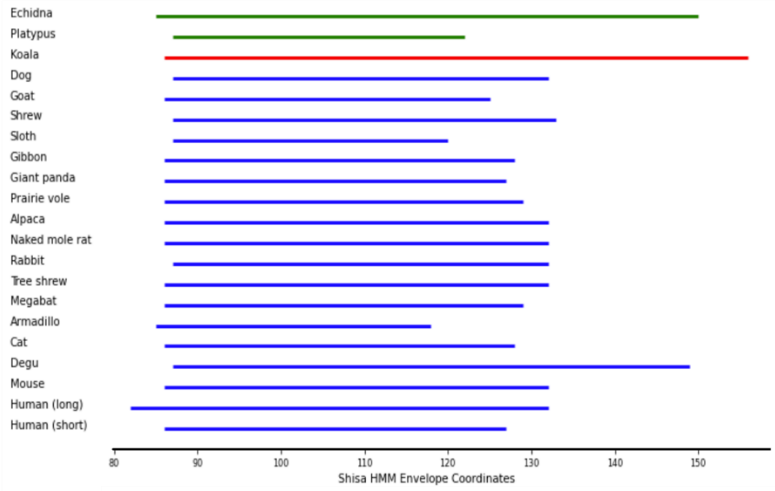

(b) Sialic acid-binding Ig-like lectin 13 (*Labeo rohita*)

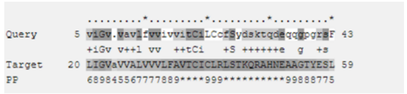

(c) Gene coagulation factor III, tissue factor (*Hagfish*)

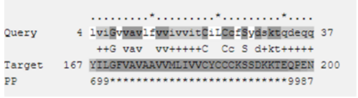

(d)

Class	Aligned	Not aligned
Mammalia	241	1
Aves	0	363

Figure S4. Inconclusive remote homology detection results. (a) Coordinates of the Shisa envelope profile that aligned to the SPAAR protein sequence. No significant results were found with queries of SPAAR in Pfam *[8]*, but many species returned Shisa family as the top result with good alignment. Four of 25 SPAAR homologs queried in Pfam did not return a Shisa result, and lengths of aligned regions varied across species with no discernable pattern, making it unconvincing as a true signal. Green lines represent monotremes, red marsupials and blue placental mammals. b) Top non-SPAAR result from hmmerscan *[9]* from Reference Proteomes database (E-value:1.5 × 10^{-7}), and (c) Ensembl genomes database (E-value: 0.0034).(d) Counts of species that aligned to human SPAAR exon coordinates in 605-way vertebrate Cactus alignment. All but one mammalian genome (gibbon) had alignments to SPAAR; no bird sequence was aligned. Though an hmmerscan query against the Reference Proteomes database returned a few significant hits that were not annotated as SPAAR, the Indian carp hit in (b) was the only significant result in fish, with no other hits in species closer in relation to placental mammals; thus, this result was inconclusive.

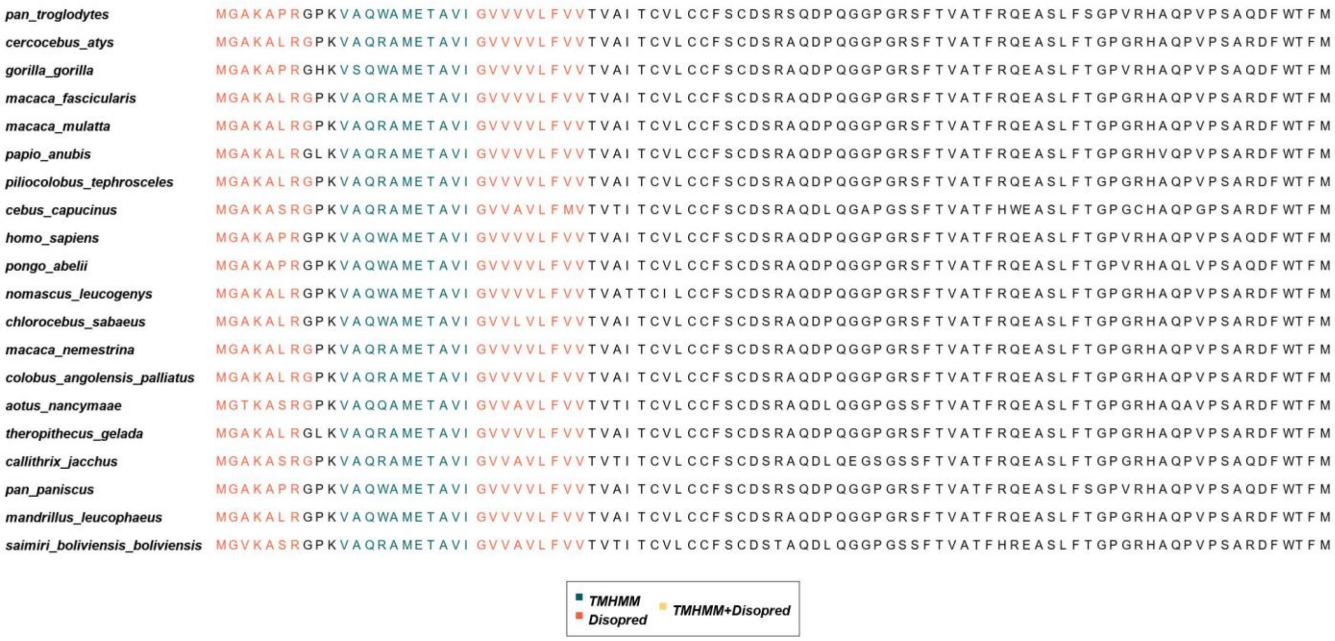

Figure S5. Prediction of transmembrane and disordered regions across primate species with long SPAAR ORF. Predicted transmembrane domains as predicted with TMHMM [10] are shown in teal, disordered residues as predicted by Disopred [11] in orange, and positions with both transmembrane domain and disorder predictions are shown in yellow.

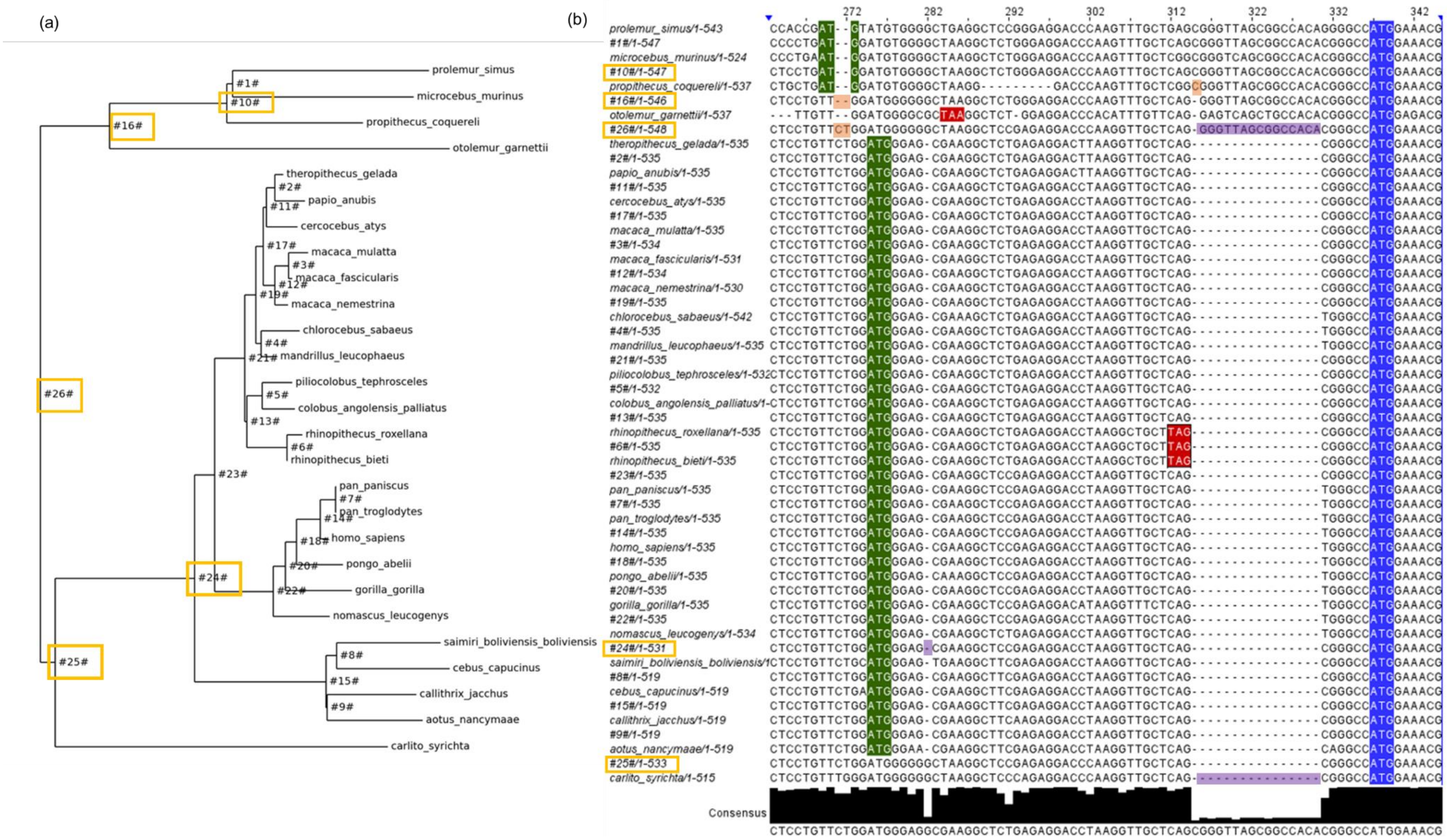

Figure S6. Ancestral reconstruction of SPAAR in primates with critical mutations leading to two elongations. (a) Phylogram of primate sequences used in ancestral sequence reconstruction, with each ancestral node labeled. Tree was taken directly from output of PRANK [12] analysis. Ancestral nodes at which critical mutations occurred are boxed in yellow. (b) Sequence alignment of primate sequences and reconstructed ancestral sequences. Start codons of the short SPAAR ORF are highlighted in blue, in-frame start codons upstream of the short SPAAR ORF are highlighted in green, and in-frame stop codons are highlighted in red. Critical mutations along the branches leading from the last common ancestor of primates to the lemur lineage are highlighted in orange, and critical mutations along branches from the last common ancestor of primates to the simian lineage are highlighted in purple.

Bibliography

1. Howe, K.L.; Achuthan, P.; Allen, J.; Allen, J.; Alvarez-Jarreta, J.; Amode, M.R.; Armean, I.M.; Azov, A.G.; Bennett, R.; Bhai, J.; Billis, K.; Boddu, S.; Charkhchi, M.; Cummins, C.; Da Rin Fioretto, L.; Davidson, C.; Dodiya, K.; El Houdaigui, B.; Fatima, R.; Gall, A.; Flicek, P. Ensembl 2021. *Nucleic Acids Res.* **2021**, *49*, D884–D891, doi:10.1093/nar/gkaa942.

2. National Library of Medicine Gene . Available online: https://www.ncbi.nlm.nih.gov/gene/ (accessed on 15 August 2021).

3. Schoch, C.L.; Ciufo, S.; Domrachev, M.; Hotton, C.L.; Kannan, S.; Khovanskaya, R.; Leipe, D.; Mcveigh, R.; O'Neill, K.; Robbertse, B.; Sharma, S.; Soussov, V.; Sullivan, J.P.; Sun, L.; Turner, S.; Karsch-Mizrachi, I. NCBI Taxonomy: a comprehensive update on curation, resources and tools. *Database (Oxford)* **2020**, *2020*, doi:10.1093/database/baaa062.

4. Altschul, S.F.; Gish, W.; Miller, W.; Myers, E.W.; Lipman, D.J. Basic local alignment search tool. *J. Mol. Biol.* **1990**, *215*, 403–410, doi:10.1016/S0022-2836(05)80360-2.

5. Robinson, J.T.; Thorvaldsdóttir, H.; Winckler, W.; Guttman, M.; Lander, E.S.; Getz, G.; Mesirov, J.P. Integrative genomics viewer. *Nat. Biotechnol.* **2011**, *29*, 24–26, doi:10.1038/nbt.1754.

6. Marin, R.; Cortez, D.; Lamanna, F.; Pradeepa, M.M.; Leushkin, E.; Julien, P.; Liechti, A.; Halbert, J.; Brüning, T.; Mössinger, K.; Trefzer, T.; Conrad, C.; Kerver, H.N.; Wade, J.; Tschopp, P.; Kaessmann, H. Convergent origination of a Drosophila-like dosage compensation mechanism in a reptile lineage. *Genome Res.* **2017**, *27*, 1974–1987, doi:10.1101/gr.223727.117.

7. Wang, Z.-Y.; Leushkin, E.; Liechti, A.; Ovchinnikova, S.; Mößinger, K.; Brüning, T.; Rummel, C.; Grützner, F.; Cardoso-Moreira, M.; Janich, P.; Gatfield, D.; Diagouraga, B.; de Massy, B.; Gill, M.E.; Peters, A.H.F.M.; Anders, S.; Kaessmann, H. Transcriptome and translatome co-evolution in mammals. *Nature* **2020**, *588*, 642–647, doi:10.1038/s41586-020-2899-z.

8. Mistry, J.; Chuguransky, S.; Williams, L.; Qureshi, M.; Salazar, G.A.; Sonnhammer, E.L.L.; Tosatto, S.C.E.; Paladin, L.; Raj, S.; Richardson, L.J.; Finn, R.D.; Bateman, A. Pfam: The protein families database in 2021. *Nucleic Acids Res.* **2021**, *49*, D412–D419, doi:10.1093/nar/gkaa913.

9. Potter, S.C.; Luciani, A.; Eddy, S.R.; Park, Y.; Lopez, R.; Finn, R.D. HMMER web server: 2018 update. *Nucleic Acids Res.* **2018**, *46*, W200–W204, doi:10.1093/nar/gky448.

10. Möller, S.; Croning, M.D.; Apweiler, R. Evaluation of methods for the prediction of membrane spanning regions. *Bioinformatics* **2001**, *17*, 646–653, doi:10.1093/bioinformatics/17.7.646.

11. Ward, J.J.; McGuffin, L.J.; Bryson, K.; Buxton, B.F.; Jones, D.T. The DISOPRED server for the prediction of protein disorder. *Bioinformatics* **2004**, *20*, 2138–2139, doi:10.1093/bioinformatics/bth195.

12. Löytynoja, A.; Goldman, N. webPRANK: a phylogeny-aware multiple sequence aligner with interactive alignment browser. *BMC Bioinformatics* **2010**, *11*, 579, doi:10.1186/1471-2105-11-579.
